# Polysaccharides From *Abrus cantoniensis* Hance Modulate Intestinal Microflora and Improve Intestinal Mucosal Barrier and Liver Oxidative Damage Induced by Heat Stress

**DOI:** 10.3389/fvets.2022.868433

**Published:** 2022-04-04

**Authors:** Ying Wang, Wenjing Sun, Enyun Wu, Kaijun Wang, Xiaogang Chen, Yao Cui, Geyin Zhang, Feifei Lv, Yuhan Wang, Xiaomin Peng, Hongbin Si

**Affiliations:** ^1^State Key Laboratory for Conservation and Utilization of Subtropical Agro-Bioresources, College of Animal Science and Technology, Guangxi University, Nanning, China; ^2^College of Biology and Pharmacy, Yulin Normal University, Yulin, China

**Keywords:** *Abrus cantoniensis* Hance, polysaccharides, heat stress, oxidative activity, Keap1-Nrf2 pathway, HPA axis, intestinal mucosal barrier, intestinal microflora

## Abstract

The protective effects of polysaccharides from *Abrus cantoniensis* Hance (ACP) on antioxidant capacity, immune function, the hypothalamus-pituitary-adrenal (HPA) axis balance, the intestinal mucosal barrier, and intestinal microflora in heat stress (HS)-induced heat-injured chickens are rarely reported. The purpose of this study was to investigate the protective effects of ACP on HS-injured chickens by enhancing antioxidant capacity and immune function, repairing the intestinal mucosal barrier, and regulating intestinal microflora. A total of 120 native roosters in Guangxi were randomly divided into 5 groups to evaluate the protective effect of ACP on chickens injured by HS (33 ± 2°C). The results showed that ACP increased the body weight and the immune organ index of heat-injured chickens, regulated the oxidative stress kinase secretion, and restored the antioxidant level of heat-injured birds. ACP significantly inhibited the secretion of corticotropin releasing hormone (CRH), adrenocorticotropic hormone (ACTH), and corticosterone (COR) and reversed the disorder of hormone levels caused by HS. ACP significantly regulated the secretion levels of immune cytokines and restored the immune function of the body. ACP significantly improved the intestinal morphology and increased the expression levels of tight junction proteins, which had a positive effect on protecting intestinal health. The results of high-throughput sequencing of the 16S rRNA gene showed that HS led to an increase in the abundance of harmful bacteria and an abnormal increase in the abundance of intestinal microflora and that ACP restored the HS-induced intestinal microflora imbalance. In conclusion, this study provides a scientific basis for ACP as an antioxidant activity enhancer to reduce liver injury, regulate intestinal microflora, and protect intestinal mucosal damage in chickens.

## Introduction

Under the environment of global warming and rising temperature, as well as the prevalence of the intensive breeding model of animal husbandry, the process of livestock and poultry breeding is often accompanied by heat stress (HS). Poultry is sensitive to high heat due to the lack of sweat glands and a high metabolic activity and other physiological characteristics, so poultry breeding is greatly affected by HS (1). HS occurs when the thermoregulation center is unbalanced due to the animal's own generation or environmental high temperature (2). A common phenomenon of HS is the imbalance between reactive oxygen species production and cellular antioxidant defense, leading to oxidative stress (3). The liver is one of the most important metabolic organs in the body, which plays an indispensable role in metabolism and detoxification. Studies have shown that the liver is more susceptible to oxidative stress in high heat (3, 4). Nuclear factor erythroid-2 related factor 2 (Nrf2) is often regarded as the sensor of an oxidant. When stimulated by oxidative stress, it binds with antioxidant response elements, thus inducing the expression of antioxidant gene transcription (5). Heat shock proteins (HSPs) can play an important role by assisting in protein folding or speeding up the degradation of misfolded proteins, in the restoration, and in the protection of the internal environment (6). HSP70 plays a key role in the adaptive response of broilers to HS by improving the antioxidant capacity, increasing digestive enzyme activities, and inhibiting the lipid peroxidation (7). Tight junction dysfunction and changes in associated intestinal integrity occur due to intestinal epithelial ischemia caused by HS (8). Intestinal barrier damage increases the permeability of the lumen antigens, which leads to inflammatory responses and stimulates the mRNA expression of pro-inflammatory cytokines (9, 10). In addition to the damage to the integrity of the intestinal barrier, the main harm of HS to the gastrointestinal tract includes changes in the microbiome (11). Studies have shown that HS can disrupt the gut flora and increases the risk of secondary infections (12). The hypothalamus is one of the important parts of the central nervous system, which has many functions such as balancing feeding, body temperature, and regulating endocrine. It can activate an emergency response to stressors in the environment (13). The hypothalamus-pituitary-adrenal (HPA) axis plays an important role in stimulating and integrating various physiological and neural responses to adverse stimuli (14). Corticosterone (COR) is a glucocorticoid secreted by the adrenal cortex and has important implications for the HPA axis. COR is a sensitive index of HS response and is related to the metabolic rate and the caloric production of broilers (13). The increase in COR indicates that the HPA system is activated in response to HS (14). Facing the severe damage caused by HS to livestock and poultry production, it is urgent to find a reasonable solution.

Farmers improve livestock and poultry resistance by strengthening feeding management and adding drugs to the livestock's diets. The excessive use of antibiotics seriously threatens the quality and safety of livestock and poultry products and the public health, and brings hidden dangers to human health, so it is particularly important to find alternatives to antibiotics (15). In recent years, Chinese herbal medicine additives have attracted widespread attention (15). Chinese herbal medicine additive is a natural substance, which can improve production performance, promote digestion and absorption, resist stress, prevent diseases, and so on (16, 17). So far, many Chinese herbal medicines have been found to contain various immune active substances, which can enhance the immune function of animals (18, 19). Studies have shown that plant extracts can improve animal performance and gut health, including barrier and absorption function, and improve antioxidant status (20).

*Abrus cantoniensis* Hance (ACP) is a Chinese herbal medicine mainly grown in Guangdong and Guangxi. It has the same origin of medicine and food. It has the effects of eliminating dampness and relieving yellowing, clearing away heat and detoxification, soothing the liver, and relieving pain (15). As early as 2005, Wong et al. proved the medicinal value of the herb as an anti-hepatitis treatment (21). The free radical scavenging activity and reducing ability of methanol extract from ACP have been proved by *in vitro* experiments (15, 22). In addition, the *in vitro* anti-tumor and immunomodulatory capabilities of ACP by *in vitro* tumor cell lines and lymphocyte proliferation experiments were demonstrated by Wu et al. (19). These studies also proved that ACP has biological functions such as antioxidant, anti-cell proliferation, and anti-bacterial activities at present, there are few reports about *in vivo* experiments using ACP. In addition, due to the climate in Guangdong and Guangxi, the probability of HS in poultry has greatly increased. ACP is rich in resources and has a good heat clearing and detoxification effect (15). Therefore, we hypothesize that the addition of ACP to the poultry's diet can effectively alleviate the adverse effects of high temperature on its liver oxidative stress, intestinal health, and body immunity.

This experiment was conducted to study the alleviating effects of ACP on liver oxidative stress, intestinal health, and the HPA axis of heat-stressed chickens to provide a theoretical basis for the development and clinical application of ACP and to lessen the effects of HS on poultry.

## Materials and Methods

### Materials and Reagents

*Abrus cantoniensis* Hance was acquired from the local market (Nanning, China). HE dye solution set were obtained from Chengdu Lilai Biological Technology Co., LTD (Chengdu, China). Chicken superoxide dismutase (SOD), malonaldehyde (MDA), Kelch-like ECH-associated protein 1 (KEAP1), Nrf2, HSP70, corticotropin releasing hormone (CRH), adrenocorticotropic hormone (ACTH), COR, secretory IgA (SIgA), interleukin 6 (IL-6), interleukin 10 (IL-10), Occludin, Claudin-1, and human tight junction protein 1 (ZO-1) ELISA kits were purchased from Jiangsu Jingmei Biotechnology Co., LTD (Jiangsu, China). All other reagents in the experiments were purchased from China and were analytically pure.

### Preparation of Polysaccharide (ACP)

*Abrus cantoniensis* Hance root pieces (1,000 g) were weighed, dried, and crushed into powder. Deionized water (1:10, w/v) was added and extracted two times at 95–100°C for 2 h. After filtration, the extracts were combined and concentrated to 1,000 ml under reduced pressure, anhydrous ethanol was added and left to stand at 4°C for 24 h. The precipitation process was repeated with anhydrous ethanol and acetone. Finally, the precipitate was freeze-dried to obtain the ACP.

### Animals and Experimental Design

All the animal experimentation was reviewed and approved by the Institutional Animal Care and Use Committee of Guangxi University (Approval No. 2021-165). Before the experiment, formaldehyde and potassium permanganate were mixed in a ratio of 2:1, the house was sealed and fumigated for 3 days, and the windows were opened for ventilation for 3 days. A total of 120 1-day-old native roosters in Guangxi were purchased from Fufeng Agriculture and Animal Husbandry Co., Ltd. and randomly divided into five treatment groups: the normal temperature control group (NHS), the high temperature model group (HS), the high temperature model_low dose group (HS_ACPL), the high temperature model_middle dose group (HS_ACPM), and the high temperature model_high dose group (HS_ACPH). There were 3 replicates in each group and 8 chickens in each replicate. The processing of daily grain includes a basal diet and an ACP supplemented diet. All chicks were treated the same on days 1–21, were provided continuous access to water and food, and were fed a basal diet at room temperature. The ingredients of the experimental diets are shown in Supplementary Table S1. The control group was kept at 23 ± 2°C from day 21 to day 42 and fed a basal diet; the HS treatment group was treated at 33 ± 2°C for 8 h (09:00–17:00), then cooled down to 23 ± 2°C for the remaining 16 h, and fed a basal diet only; the treatment group was given the same heat treatment as the HS group for 8 h a day, and the diet was, respectively, supplemented with 200, 400, and 600 mg/kg of ACP (Table 1). In addition, all chickens were given 24 h of light at 1–3 days of age and then decreased by 3 h per week until natural light was reached.

**Table 1:** Different treatment groups in chickens.

**Group**	**Treatment**	**Duration**
NHS	basal diet (normal temperature)	Days 1–42
HS	basal diet (normal temperature)	Days 1–21
	basal diet (high temperature)	Days 21–42
HS_ACPL	basal diet (normal temperature)	Days 1–21
	add 200 mg/kg ACP (high temperature)	Days 21–42
HS_ACPM	basal diet (normal temperature)	Days 1–21
	add 400 mg/kg ACP (high temperature)	Days 21–42
HS_ACPH	basal diet (normal temperature)	Days 1–21
	add 600 mg/kg ACP (high temperature)	Days 21–42

*NHS, normal temperature control group; HS, heat stress; HS_ACPL, treatment-low dose group with 200 mg/kg ACP; HS_ACPM, treatment-middle dose group with 400 mg/kg ACP; HS_ACPH, treatment-high dose group with 600 mg/kg ACP*.

### Sample Collection

On the 42nd day of the experiment, after 8 h of starvation treatment, weighed and euthanized after anesthesia. Blood samples were collected from the jugular vein, and the serum was separated and then, cryopreserved for subsequent study. The bursa of Fabricius and the spleen were collected and weighed, and the liver tissue was collected and stored at −80°C. The collected cecal intestinal contents, the cecum, and the ileum intestines were stored at −80°C for subsequent testing. Part of the ileum specimens were immersed in a 4% formalin solution, and further histological analysis was performed after fixation for 24 h.

### Determination of Body Weight and Organ Indices

The chickens in each group were weighed and sacrificed at the end of the experiment. The bursa of Fabricius and the spleen were removed and weighed. The organ index was calculated according to the following formula: organ index (mg/g) = weight of spleen or bursa of Fabricius (mg)/chicken body weight (g).

### Evaluation of Oxidative Stress Damage and Anti-Oxidative Stress

Through the determination of the levels of antioxidant enzymes in the serum and liver tissues, the local oxidative stress and the anti-oxidative stress of the body and the liver can be evaluated. The levels of SOD, MDA, KEAP1, Nrf2, and HSP70 in the serum and the liver tissues were determined according to the instructions of the Jiangsu Jingmei ELISA Kit.

### Hypothalamic-Pituitary-Adrenal Axis

Determination of the HPA axis related hormone levels in serum and ileum. We took 1 g ileum tissue, added 9 g of PH7.2-7 PBS, and homogenized the specimen fully. The sample was centrifuged for 20 min (2,000 rpm), and the supernatant was collected carefully and separated for testing. CRH, ACTH, and COR levels in the serum and the ileum tissues homogenate supernatants that were determined using the Jiangsu Jingmei ELISA kit.

### Determination of Cytokine Levels

The levels of cytokines in the serum and the ileum tissues were determined by ELISA. The levels of SIgA, IL-6, and IL-10 in the serum and ileum tissue homogenate supernatant were determined by the ELISA kit.

### Intestinal Tight Junction Protein

The levels of Occludin, Claudin-1, and ZO-1 in the ileum tissue were determined by the supernatant of the ileum tissue homogenate. The test method was carried out in accordance with the instructions provided by the ELISA manufacturer.

### Histological Examination of Ileum

By a series of concentrations of ethanol treatment, the collected ileum tissue was fixed in a 10% formaldehyde solution for 24 h to achieve a better dehydration effect. Then, it was processed into paraffin-embedded blocks and cut into slices with a thickness of 5 μm. The slices were treated with xylene and series concentration ethanol, stained with hematoxylin-eosin, and sealed with a neutral gum to observe the histological changes of the ileum. The image acquisition of slices was done using BA210Digital (McDid Industrial Group Co., Ltd.) and a digital three-eye camera micro camera system (40 × magnification). Motic Images Advanced 3.2 Analysis software collected images and measured the height and crypt depth of intact villi. The ratio of villus height (VH) to crypt depth (CD) was calculated as an indicator of small intestine injury.

### Genomic DNA Extraction and 16S-rDNA Sequencing of Feces

DNA in the cecum content was extracted according to the method in the DNA Kit (Omega Bio-tek, Norcross, GA, USA). The purity and the quality of the genomic DNA were checked on 0.8% agarose gel electrophoresis and high-throughput sequencing. The extracted DNA samples were detected by 1% agarose gel electrophoresis and spectrophotometry (260/280 nm optical density ratio) for quality inspection and high-throughput sequencing. The V3-4 hyper-variable region of the bacterial 16S rRNA gene were amplified with the primers 338F (ACTCCTACGGGAGGCAGCAG) and 806R (GGACTACHVGGGTWTCTAAT). The PCR was carried out on a Mastercycler Gradient (Eppendorf, Germany) using 25 μl reaction volumes; three PCR products per sample were pooled to mitigate reaction-level PCR biases. The PCR products were purified using a QIAquick Gel Extraction Kit (QIAGEN, Germany), quantified using Real Time PCR, and sequenced. Deep sequencing was performed on the Miseq platform at Allwegene Company (Beijing, China). After the run, image analysis, base calling, and error estimation were performed using Illumina Analysis Pipeline Version 2.6 (Illumina, Inc., San Diego, CA, USA). The raw data were first screened and the sequences were removed from consideration if they were shorter than 200 bp with a low-quality score (20), contained ambiguous bases, or did not exactly match to primer sequences and barcode tags. Qualified reads were separated using the sample-specific barcode sequences and trimmed with Illumina Analysis Pipeline Version 2.6. Then, the dataset was analyzed using QIIME. The sequences were clustered into operational taxonomic units (OTUs) at a similarity level of 97% to generate rarefaction curves and to calculate the richness and diversity indices. The Ribosomal Database Project (RDP) Classifier tool was used to classify all sequences into different taxonomic groups (http://rdp.cme.msu.edu/classifier/classifier.jsp). To examine the similarity between different samples, clustering analyses and PCA were used based on the OTU information from each sample using R. The dominance of bacterial communities between groups were analyzed by the linear discriminant analysis [LDA, LDA score (Log10) = 4 as the boundary value] with LEfSe effect size.

### Statistical Analyses

All experimental data in the chart were presented as mean ± standard deviation, and three replicate experiments were carried out. Duncan's multiple range test and the least significant difference (LSD) test were used to evaluate the differences between groups. Statistical analysis was performed using SPSS software 26 (SPSS Inc., Chicago, IL, USA); *P* < 0.05 was considered statistically significant.

## Results

### Effect of ACP on Body Weight and Immune Organ Indices

**Figure 1:**
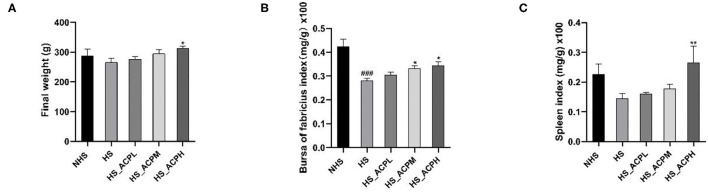
Effects of *Abrus cantoniensis* Hance (ACP) supplementation on body weight and immune organ indices. **(A)** Final weight, **(B)** The bursa of Fabricius index, **(C)** The Spleen index. Data are presented as the mean ± SEM. ^###^*p* < 0.001 compared with the normal temperature control (NHS) group; *p < 0.05, **p < 0.01 compared with the heat stress (HS) group.

The body weight, the bursa of Fabricius, and the spleen index of chickens are shown in Figure 1. Under high temperature, the body weight of chickens had a tendency to decrease compared with the NHS group. ACP in the low, medium, and high dose groups had a dose-dependent effect on body weight gain, and the effect in the HS_ACPH group was the most significant (*P* < 0.05). The bursa of the Fabricius index in the HS group was significantly lower than that in the NHS group (*P* < 0.001) and in the HS_ACPL, HS_ACPM, and HS_ACPH groups, and the bursa of Fabricius index increased in a dose-dependent manner, especially in the HS_ACPM and HS_ACPH groups (*P* < 0.05). However, the spleen index of the HS_ACPH group was significantly higher than that of the HS group (*P* < 0.05). This study indicates that ACP improved HS-induced weight loss in birds and prevented the atrophy of the body's immune organs; the HS_ACPH group had the best effect.

### Effects of ACP on Oxidative Stress Kinase and Related Proteins Levels in the Serum and Liver Tissues

**Figure 2:**
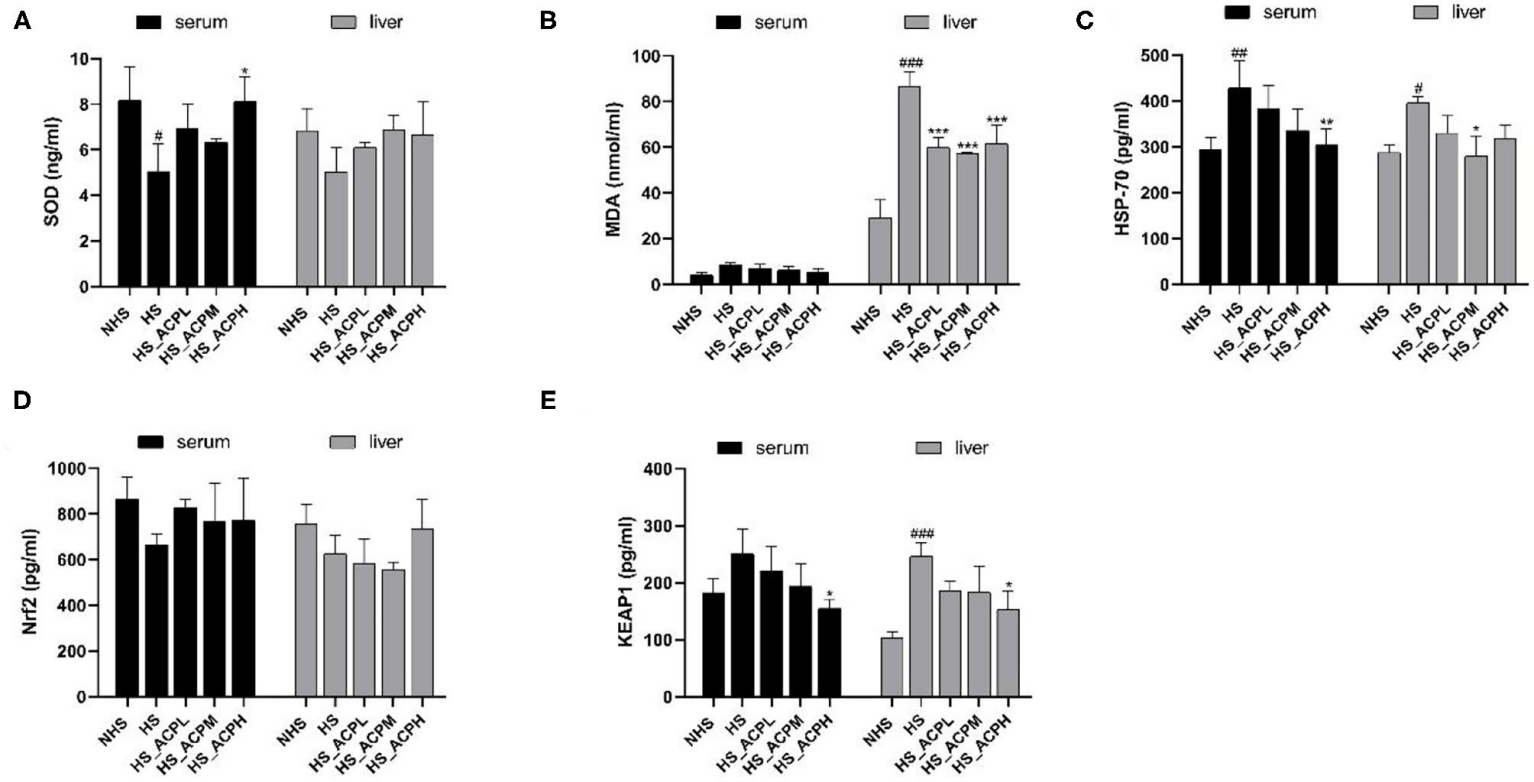
Effects of ACP supplementation on the levels of antioxidants related in serum and liver. **(A)** superoxide dismutase (SOD), **(B)** malonaldehyde (MDA), **(C)** heat shock protein 70 (HSP-70), **(D)** nuclear factor erythroid-2 related factor 2 (Nrf2), **(E)** Kelch-like ECH-associated protein 1 (Keap1). Data are presented as the mean ± SEM. ^#^*p* < 0.05, ^##^*p* < 0.01, ^###^*p* < 0.001 compared with the NHS group; *p < 0.05, **p < 0.01, ***p < 0.001 compared with the HS group.

By measuring the levels of oxidative stress-related enzymes in poultry serum and liver tissues, the regulatory effect of ACP on poultry anti-oxidative stress was evaluated. As shown in Figure 2A, compared with the NHS group, the serum SOD level in the HS group significantly decreased but significantly increased in the HS_ACPH group (*P* < 0.05). The SOD level in the liver tissue of the five groups showed an upward trend in all three dose groups compared to the HS group. There was no statistically significant difference in serum MDA levels among the groups. However, in liver tissues, MDA levels in the HS group were significantly higher than those in the NHS group (*P* < 0.001), while MDA levels in the three treatment groups were lower than those in the HS group (*P* < 0.001) (Figure 2B). In the serum tissue, the HSP70 level in the HS group was significantly higher than that in the NHS group (*P* < 0.01); in the three treatment groups, the HSP70 level decreased in a dose-dependent manner, especially in the HS_ACPH group (*P* < 0.01). In liver tissue, the level of HSP-70 in the HS group was also significantly higher than that in the NHS group (*P* < 0.05) and that in the HS_ACPM group was significantly lower compared with the HS group (*P* < 0.05) (Figure 2C). In the serum and liver tissues, the level of Nrf2 in the HS group was lower than that in the NHS group, and this phenomenon was ameliorated by ACP (Figure 2D). Compared with the HS group, the KEAP1 levels in the serum tissue in the three treatment groups showed a dose-dependent downward trend. In liver, the KEAP1 levels in the NHS group and the HS_ACPH group were significantly lower than that in the HS group (*P* < 0.001, *P* < 0.05) (Figure 2E). As shown in Figure 2, the changes of oxidative stress kinases and their related proteins in the serum and liver tissues of all experimental groups were consistent. By comparing the HS group with the NHS group, it was found that HS did affect the oxidative activity related indexes of the body. On the other hand, the above effects caused by HS can be ameliorated by ACP, which increased the levels of SOD and Nrf2 and decreased the levels of MDA, Keap1, and HSP70.

### Effect of ACP on the HPA Axis in the Serum and the Ileum

**Figure 3:**
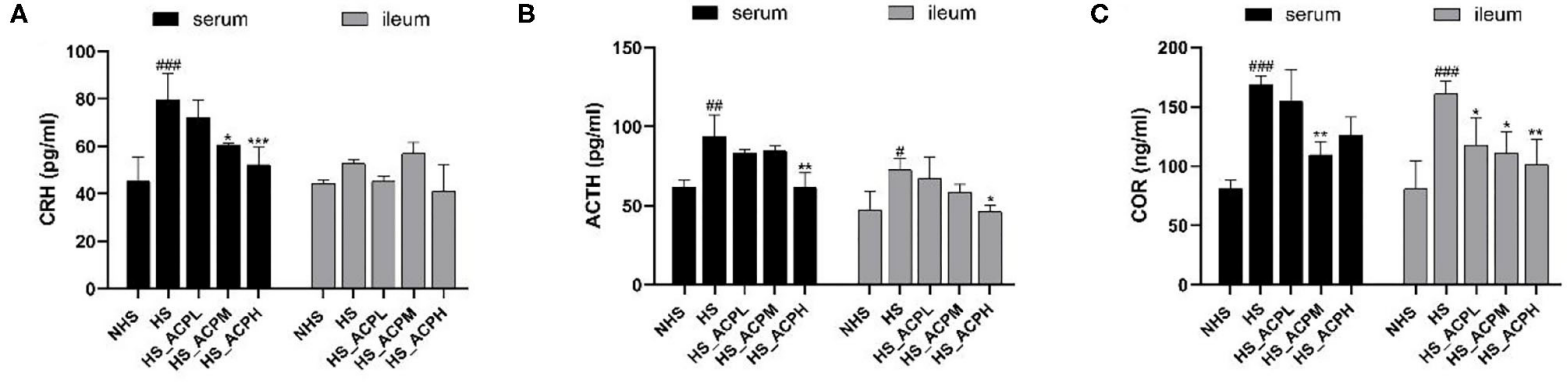
Effects of ACP supplementation on the levels of hypothalamus-pituitary-adrenal (HPA) axis in the serum and ileum. **(A)** corticotropin releasing hormone (CRH), **(B)** adrenocorticotropic hormone (ACTH), **(C)** corticosterone (COR). Data are presented as the mean ± SEM. ^#^*p* < 0.05, ^##^*p* < 0.01, ^###^*p* < 0.001 compared with the NHS group; *p < 0.05, **p < 0.01, ***p < 0.001 compared with the HS group.

As shown in Figure 3, in the serum tissue, compared with the NHS group, the levels of CRH, ACTH, and COR in the HS group showed a significantly upward trend (*P* < 0.001, *P* < 0.01, *P* < 0.001). Compared with the HS group, the level of CRH decreased in a dose-dependent manner, and the effect was significant in the HS_ACPM and HS_ACPH groups (*P* < 0.05, *P* < 0.001); the ACTH level in the HS_ACPH group and the COR level in the HS_ACPM group significantly decreased (*P* < 0.01). In the ileum tissue, there was no significant change in CRH levels; the ACTH and COR levels in the HS group were significantly higher than those in the NHS group (*P* < 0.05, *P* < 0.001); compared with the HS group, both ACTH and COR showed a significant dose-dependent downward trend (*P* < 0.05, *P* < 0.01). The results showed that the balance of HPA was disrupted by HS, which was reversed by adding ACP to increase the level of related hormones.

### Effects of ACP on Cytokines and Intestinal Tight Junction Protein Levels

**Figure 4:**
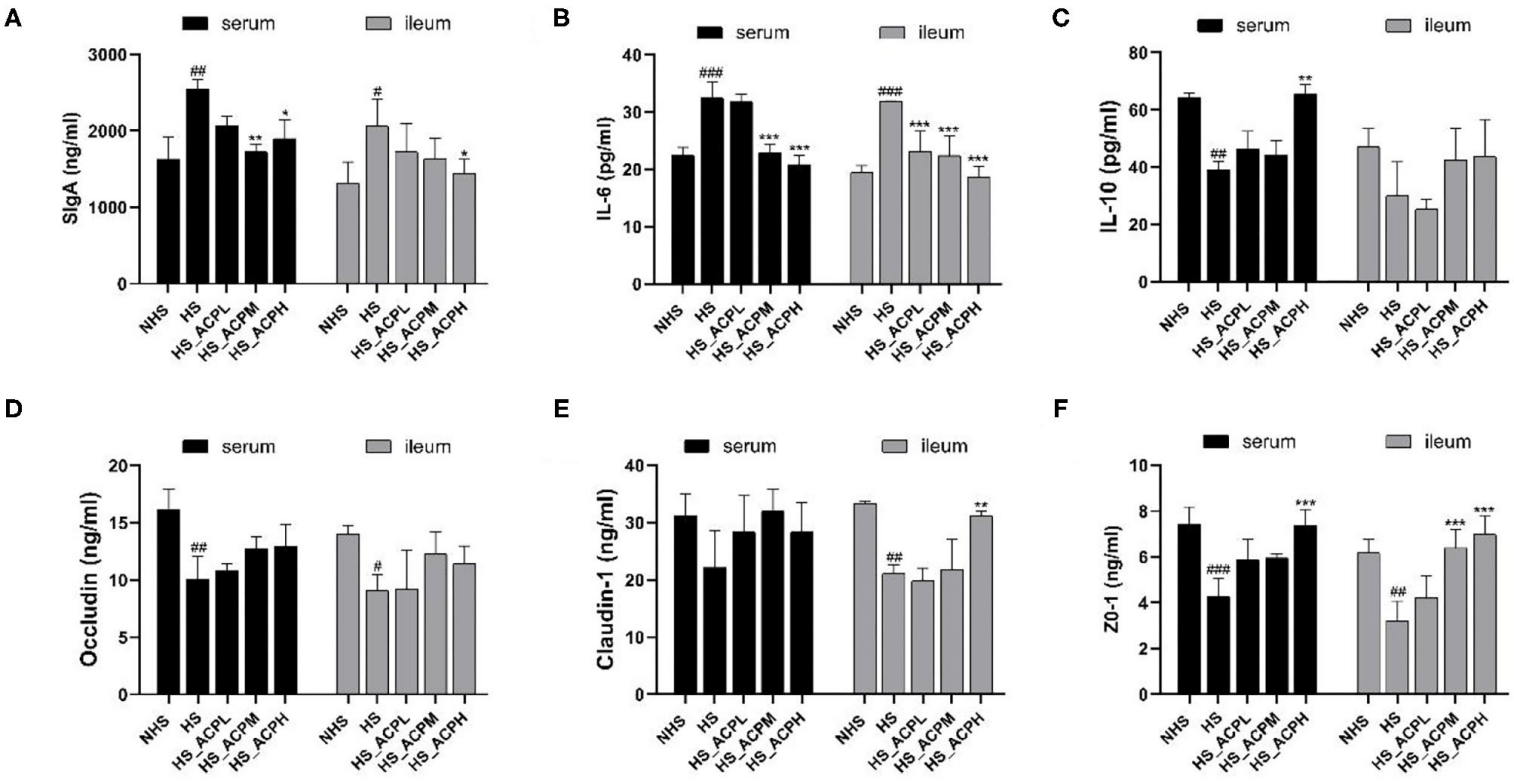
Effects of ACP supplementation on the levels of immune factors in the serum and the ileum, and tight junction proteins. **(A)** secretory IgA (SIgA), **(B)** interleukin 6 (IL-6), **(C)** interleukin 10 (IL-10), **(D)** Occludin, **(E)** Claudin-1, **(F)** human tight junction protein 1 (ZO-1). Data are presented as the mean ± SEM. ^#^*p* < 0.05, ^##^*p* < 0.01, ^###^*p* < 0.001 compared with the NHS group; *p < 0.05, **p < 0.01, ***p < 0.001 compared with the HS group.

The results, as shown in Figures 4A,B, in the serum, SIgA, and IL-6 levels were significantly lower not only in the NHS group (*P* < 0.01, *P* < 0.001) but also in the HS_ACPM (*P* < 0.01, *P* < 0.001) and HS_ACPH groups (*P* < 0.05, *P* < 0.001) than in the HS group. In the ileum, the trend of SIgA and IL-6 levels was similar to that in the serum. However, in the serum, the IL-10 level in the NHS and HS_ACPH groups was significantly higher than that in the HS group (*P* < 0.01) (Figure 4C). The results showed that the immune activity of the body was suppressed by HS, while this immune damage was repaired by ACP by decreasing IL-6 and increasing IL-10 levels. It was not difficult to see that Occludin and ZO-1 levels in the serum of the HS group were significantly lower than those of the NHS group (*P* < 0.01, *P* < 0.001). The Claudin-1 levels were not significant, and it was confirmed that HS caused a certain damage to the ileum permeability of chickens. Compared with the HS group, ACP supplementation had a certain improvement effect, and the level of ZO-1 in the HS_ACPH group significantly increased (*P* < 0.001). The levels of Occludin, Claudin-1, and ZO-1 in ileum in the HS group were significantly lower than those in the NHS group (*P* < 0.05, *P* < 0.01). The Claudin-1 level was significantly higher in the HS_ACPH group than in the HS group (*P* < 0.01). The level of ZO-1 increased in a dose-dependent manner, and the effect was very significant in the HS_ACPM and HS_ACPH groups (*P* < 0.001) (Figures 4D–F). In summary, the damage of intestinal permeability caused by HS was restored by ACP.

### Ileum Histomorphology

**Figure 5:**
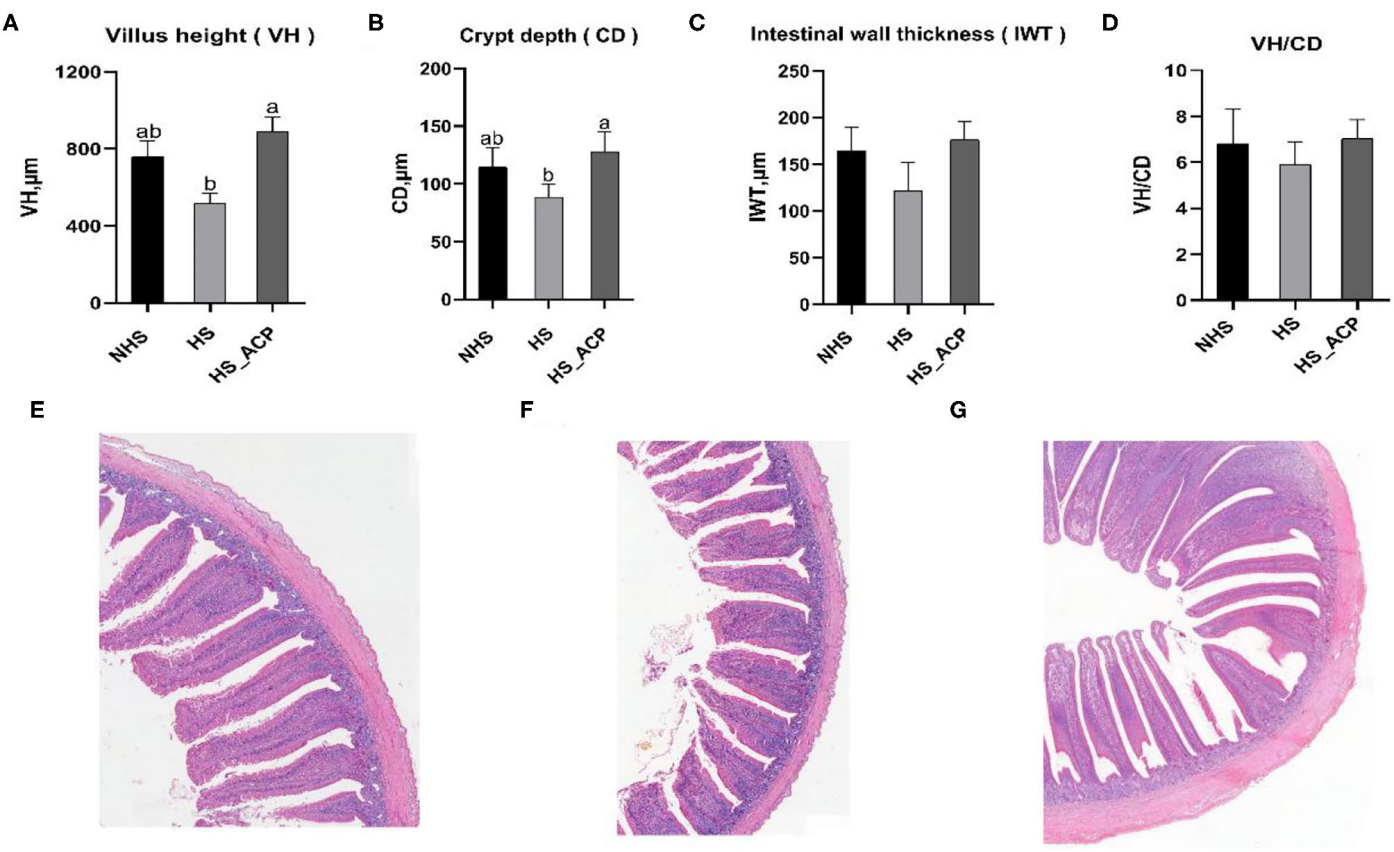
Effects of ACP supplementation on the ileum histomorphology of the heat-stressed broilers. **(A)** Villus height (VH), **(B)** Crypt depth (CD), **(C)** Intestinal wall thickness (IWT), **(D)** VH: CD, **(E)** Ileum tissue section of the NHS group, **(F)** Ileum tissue section of the HS group, and **(G)** Ileum tissue section of the high temperature model group with ACP dose HS_ACP group. The effect of the treatment was statistically different at *p* < 0.05. Different letters indicate the significant difference between treatments.

To further elucidate the protective effect of ACP on the intestinal barrier, we performed a histological analysis of the chicken ileum. Preliminary results showed that HS_ACPH group had the best overall effect among the three dosage groups, so we chose HS_ACPH group for further study. The subgroups in the further study were the NHS, HS and HS_ACPH groups (hereinafter renamed the HS_ACP group). The VH and CD of the HS group were lower than those in the NHS group, which indicated that the ileum structure was destroyed by HS. Dietary supplementation of ACP significantly increased VH and CD, indicating that intestinal damage caused by HS was repaired by ACP (*P* < 0.05) (Figures 5A,B). At the same time, although intestinal wall thickness (IWT) and VH/CD in the ACP group had no statistical significance compared with the HS group, the addition of ACP made IWT and VH/CD return to normal (*P* >.05) (Figures 5C,D). These results were more intuitively verified in ileum tissue sections (Figures 5E–G).

### Alpha and Beta Diversity of Cecal Microbiota

**Figure 6:**
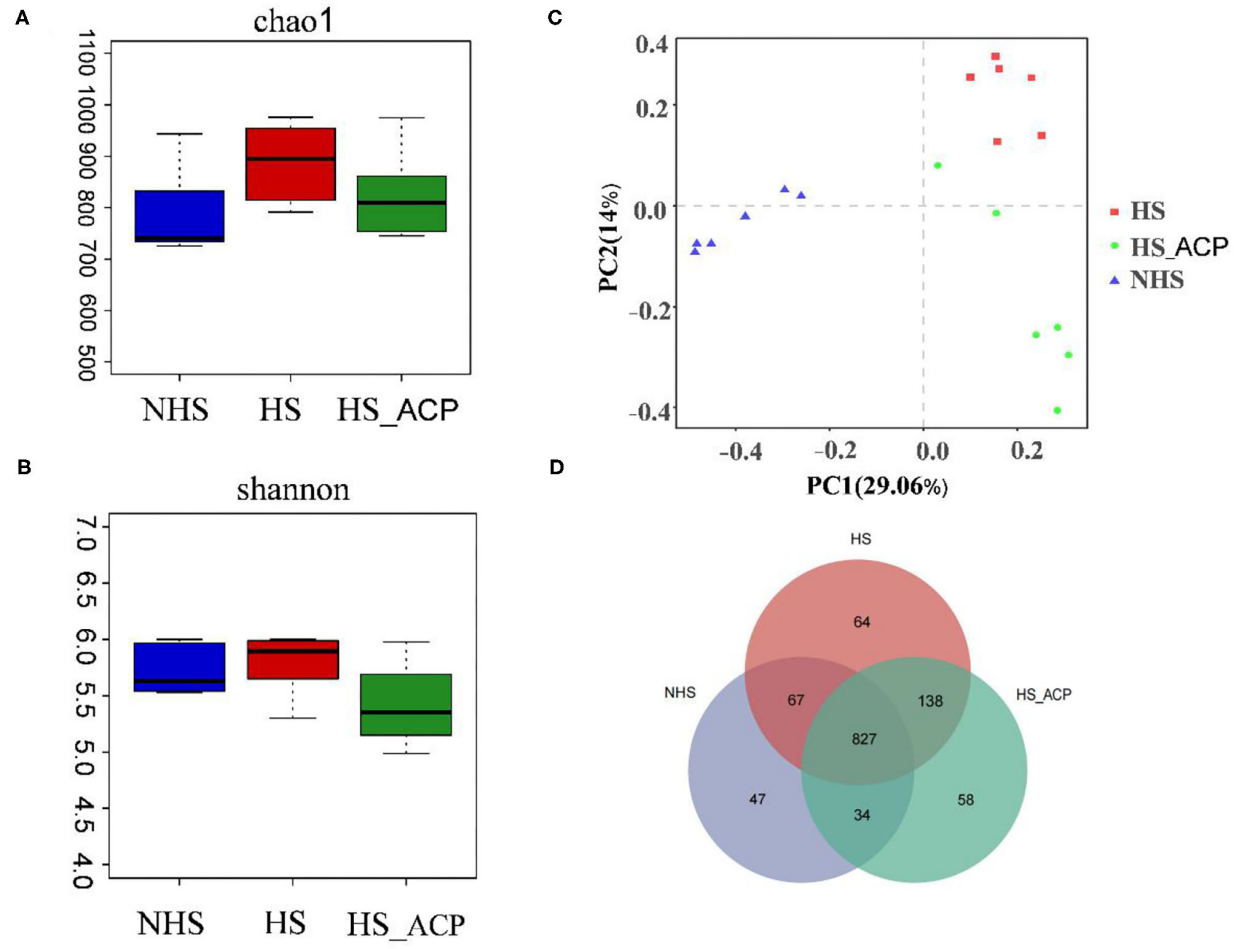
Effect of adding ACP on microbial diversity in heat-stressed broilers. **(A)** Chao1 index, **(B)** Shannon entropy, **(C)** Principal component analysis of gut microbiota, **(D)** Similarity and difference of bacteria compositions in different experimental groups at the operational taxonomic unit (OTU) level.

In this study, 16S-RDNA of bacterial DNA in the cecal contents of poultry was detected by high-throughput gene sequencing to clarify the regulatory effect of ACP on intestinal flora. The results of the rarefaction curve and the Shannon-Wiener curve showed that most of the bacteria could be captured (Supplementary Figures S1, S2). We used Chao1 and Shannon indices to evaluate the richness and diversity of the intestinal microbiome. The result shows that the Chao1 index of the HS group is higher than that of the NHS group. It was beneficial that ACP reversed this phenomenon (Figure 6A), and the Shannon index showed no significant change in the three treatment groups (Figure 6B). To distinguish differences in the microbial community structure, we calculated the diversity of microbial composition based on the principal component analysis (PCA) (Figure 6C). The cecal microflora of the HS, NHS and ACP groups were completely separated.

Differences in community composition between samples of different treatment groups were further investigated using a variety of visualization methods. By drawing a Venn diagram, the common richness of intestinal microflora in each group was analyzed. As shown in the Venn diagram, a total of 827 OTUs overlapped among the three groups, 975 OTUs were found in the NHS group, 1,096 OTUs in the HS group, and 1,057 OTUs in the ACP group (Figure 6D).

### Microbial Composition

**Figure 7:**
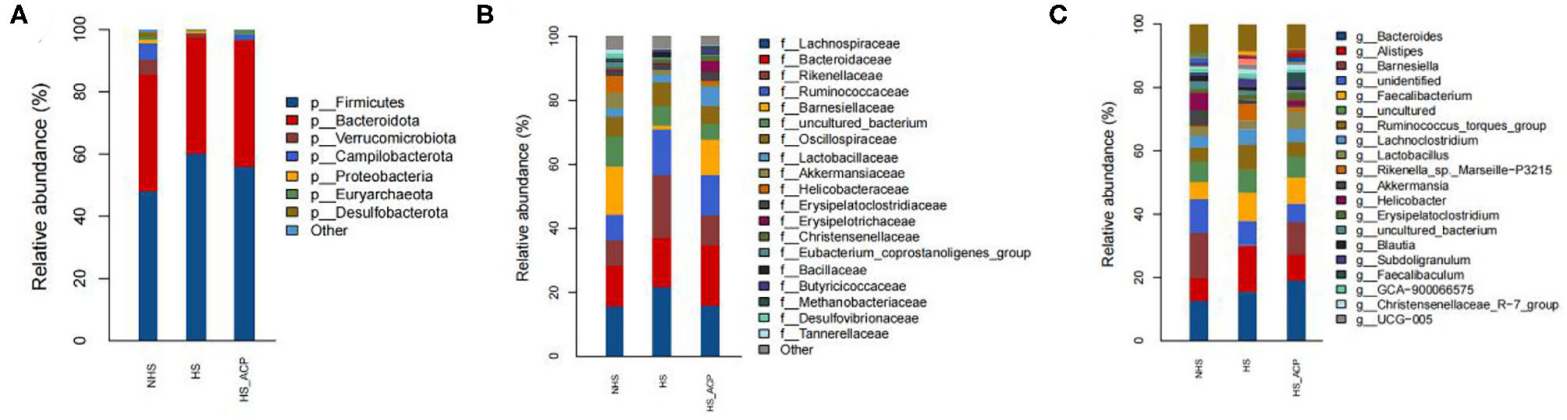
Relative abundance of microbiota at the **(A)** Phylum, **(B)** Family, and **(C)** Genus level.

At the phyla level (Figure 7A), *Firmicutes* and *Bacteroidetes* accounted for the largest proportion, especially in the HS (97%), ACP (96%), and NHS (83%) groups. The proportion of *Firmicutes* in the HS group was 60%, which was higher than that in the NHS group (25%) (*P* < 0.05). The ACP group decreased the abundance of *Firmicutes* and increased the abundance of *Bacteroidetes*. At the family level (Figure 7B), the top five bacteria abundance ratios were *Lachnospiraceae, Bacteroidaceae, Rikenellaceae, Ruminococcaceae*, and *Barnesiellaceae*. Among them, there were significant differences in the abundance of *Rikenellaceae* and *Barnesiellaceae* in the three groups as follows: compared with the NHS group, the abundance of *Rikenellaceae* in the HS group significantly increased; compared with the HS group, the HS_ACP group showed significantly lower *Rikenellaceae* abundance to the NHS group (*P* < 0.05). On the contrary, *Barnesiellaceae* abundance in the HS group was significantly lower than that in the NHS and HS_ACP groups (*P* < 0.05). At the genus level (Figure 7C), *Bacteroidotes, Alistipes*, and *Barnesiella* accounted for a large proportion. The abundance of *Alistipes* in the HS group was significantly higher than that in the NHS; compared with the HS group, the ACP treatment significantly reduced the abundance of *Alistipes* in the HS group and tended to return to a normal level (*P* < 0.05). The abundance of *Barnesiella* in the HS group was significantly lower than that in the NHS and HS_ACP groups (*P* < 0.05), and there was no difference between the two groups. A comparative analysis of the abundance of dominant bacteria at each level in the three groups is shown in Supplementary Table 2.

### Analysis of Dominant Bacteria

**Figure 8:**
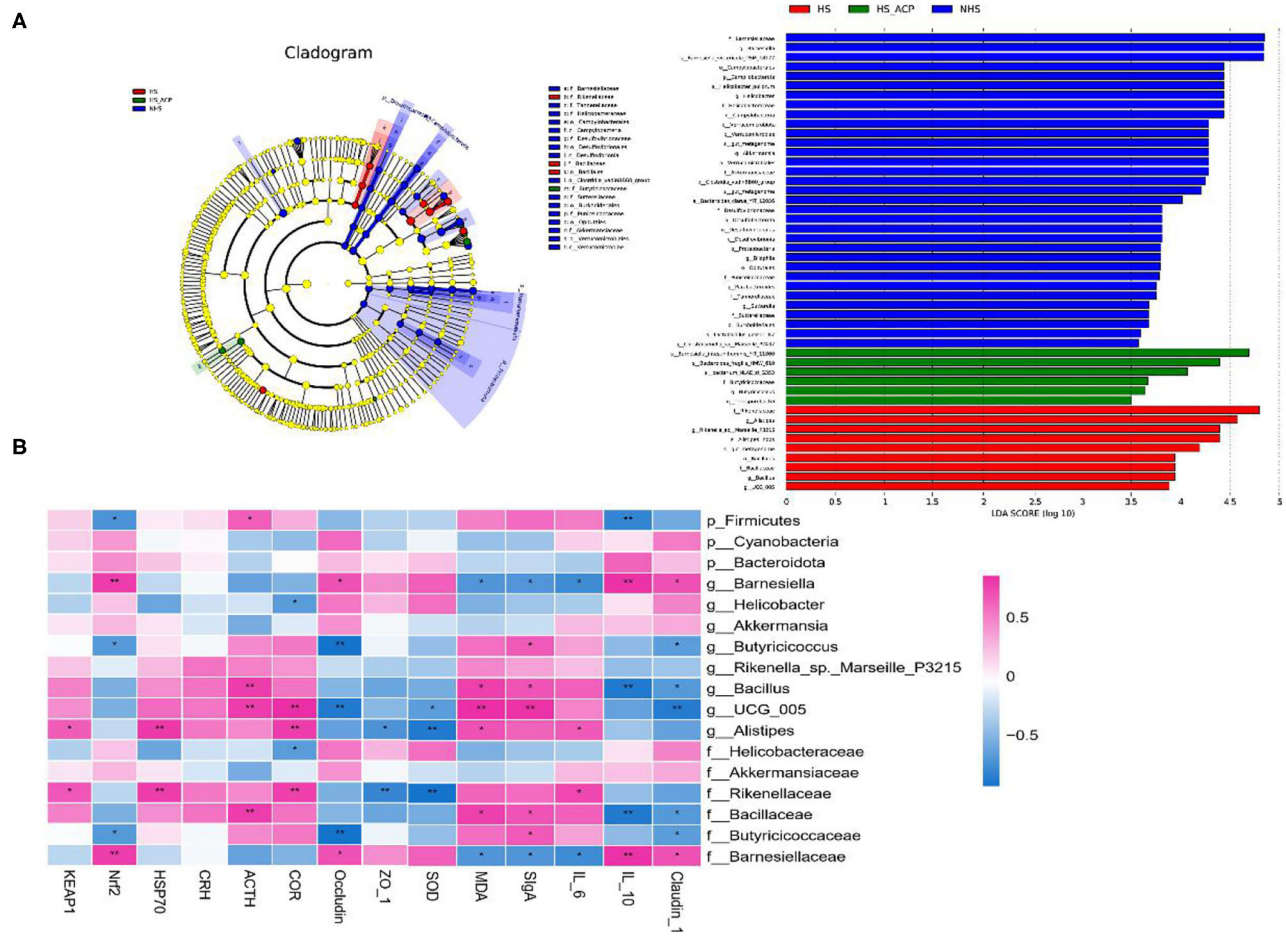
**(A)** Difference in dominant microorganisms among each group *via* cladogram and distribution histogram based on linear discriminant analysis (LDA). Only the results meeting an LDA significant threshold of >3.5 were shown. The greater the LDA score, the more significant the phylotype microbiota was in the comparison, **(B)** A heatmap showing the Spearman correlation coefficient between several significant enriched intestinal bacteria and measurement parameters. Red cells represent positive correlation and blue cells represent negative correlation. *p < 0.05, **p < 0.01.

The LEfSe analysis was used to compare the high-dimensional categories, and significant differences were found in the bacterial community dominance among the three groups. According to the results (Figure 8A), *Barnesiella* (from the family *Barnesiella* to the species), *Campylobacter* (from the order *Campylobacter* to the class), *Helicobacter* (from the family *Helicobacte*r to the species), and *Verrucomicrobiae* (from the phylum *Verrucomicrobiae* to the order) showed a higher LDA score, indicating a large number of OTUs in the NHS group. *Rikenellaceae* (from the family *Rikenellaceae* to the genus), *Alistipes* (from the genus *Alistipes* to the species), and *Bacillales* (from the order *Bacillales* to the genus) had higher LDA scores, indicating that they were the key flora types which led to cecal microflora imbalance in the HS group. Compared with the HS group, the abundance of *Barnesiella* and *S_Bacteroides* in the ACP group recovered to a higher level, similar to that in the NHS group. These results suggest that the ACP intervention improved the intestinal microbiota imbalance and promoted the proliferation of specific bacteria in the HS-induced poultry to a certain extent.

### Correlation Between Different Microbial Compositions and Measured Parameters

The Spearman correlation analysis was used to determine the potential relationship between different microbiota and the parameters involved in the study (Figure 8B). Antioxidants related to SOD and Nrf2 positively correlated with the phylum *Bacteroidota*, the genera *Barnesiella* and *Helicobacter*, the family *Barnesiellaceae* and *Helicobacteraceae* but negatively correlated with the phylum *Firmicutes*, the genera *Butyricicoccus, Bacillus*, and UCG_005, and the family *Rikenellaceae, Bacillaceae*, and *Butyricicoccaceae*. Coincidentally but differently, MDA, HSP70, and Keap1 were also associated with these microorganisms, but in reverse order. However, there seems to be no obvious correlation between Keap1 and the genus *Butyricicoccus* and between HSP70 and the phylum *Cyanobacteria*. The CRH, ACTH, and COR hormone-associated flora in the HPA axis are similar as there was a positive correlation with the phylum *Firmicutes*, the genera *Butyricicoccus, Rikenella_*sp*._Marseille_P*3215*, Bacillus*, UCG_005, and *Alistipes*, the family *Rikenellaceae, Bacillaceae*, and *Butyricicoccaceae*, and a negative correlation with the phyla *Cyanobacteria* and *Bacteroidota*, the genera *Barnesiella, Helicobacter*, and *Akkermansia*, and the family *Helicobacteraceae, Akkermansiaceae*, and *Barnesiellaceae*. A difference is that CRH is not sensitive to the phyla *Cyanobacteria* and *Bacteroidota*, the genera *Barnesiella* and *Butyricicoccus*, the family *Butyricicoccaceae* and *Barnesiellaceae*. COR has no correlation with the phylum *Bacteroidota*. The anti-inflammatory cytokine, IL-10, positively correlated with the phylum *Bacteroidota*, the genus *Barnesiella*, and the family *Barnesiellaceae* but negatively correlated with the phylum *Firmicutes*, the genera *Butyricicoccus, Rikenella*_sp*._Marseille_P*3215*, Bacillus*, UCG_005, and *Alistipes*, and the family *Rikenellaceae, Bacillaceae*, and *Butyricicoccaceae*. Coincidentally, but differently, pro-inflammatory cytokines, IL-6 and SIgA, are also potentially related to these microorganisms, while the association is opposite to IL-10. The tight junction proteins, Occludin and Claudin-1, positively correlated with the phyla *Cyanobacteria* and *Bacteroidota*, the genera *Barnesiella* (*P* < 0.05), *Helicobacter*, and *Akkermansia*, and the family *Helicobacteraceae, Akkermansiaceae*, and *Barnesiellaceae* (*P* < 0.05) but negatively correlated with the phylum *Firmicutes*, the genera *Butyricicoccus* (*P* < 0.05), *Bacillus* (*P* < 0.05), UCG_005 (*P* < 0.01), *Rikenella_*sp*._Marseille*_P321*5*, and *Alistipes*, and the family *Rikenellaceae, Bacillaceae*, (*P* < 0.05), and *Butyricicoccaceae* (*P* < 0.05). In short, intestinal microbes are closely related to oxidative stress, HPA axis regulation, and intestinal health.

## Discussion

Environmental HS caused negative effects by inducing oxidative stress and hormone secretion disorder in the hypothalamus and the intestinal barrier (23, 24). The screening of suitable antioxidant additives is an effective way to reduce heat damage. In this study, 200, 400, and 600 mg/kg ACP feed were added to observe its alleviating effect to HS-induced injury in poultry. Spleen is an important immune organ, and the bursa of Fabricius is a unique immune organ of birds. The immune organ index is a key index to describe the function of immune organs (25). Some studies have shown that HS reduced the immune organ index (26). Studies have reported that polysaccharides have good immune enhancement ability, and HLP treatment increased the immune organ index (27). Our results are consistent with previous studies; HS reduced body weight and the immune organ index, and the addition of ACP lessened this phenomenon, suggesting that ACP enhances host immune function by stimulating the development of immune organs. Dietary ACP supplementation significantly improved the antioxidant activity of heat-stressed birds. This is consistent with the results of previous studies (28). SOD is an important member of antioxidant enzymes in the biological system, which can catalyze the superoxide anion radical disproportionation to generate oxygen and hydrogen peroxide, and plays a crucial role in the balance between oxidation and anti-oxidation. MDA is a product of lipid peroxidation and is sensitive to oxidative stress. SOD levels in the serum and the liver significantly decreased, and MDA levels significantly increased, under high temperature stimulation, compared with the NHS group. This is consistent with the results of previous studies (29). Compared with the HS group, the SOD level in the HS_ACP group significantly increased and the MDA level significantly decreased after ACP intervention. These results indicated that the damage of HS on the antioxidant capacity of poultry was significantly lessened by ACP. In addition, the Spearman correlation revealed that SOD was negatively correlated with g_UCG_005*, g_Alistipes*, and *f_Rikenellaceae* and positively correlated with *Barnesiella*. However, the correlation between the above microflora and MDA was opposite. Interestingly, the LEfSe analysis found that the first three microflora were mainly enriched in the HS group, while the last one was mainly enriched in the HS_ACP group. In conclusion, these findings suggest that ACP improves antioxidant capacity by balancing the secretion of oxidative reactive kinase, which may be related to intestinal microbes.

Keap1-Nrf2 and HSPs are important endogenous protective mechanisms of HS initiation, participating in the regulation of antioxidant enzymes and playing a dual protective role in cell adaptation and survival (30). Nrf2 is a key factor in the Keap1-Nrf2 pathway, which plays an important role in activating antioxidant signaling pathways to prevent oxidative stress-induced cell and tissue damage and to maintain redox balance (31); it can also boost the production of other antioxidants (32); Keap1 is a negative regulator of Nrf2 (33, 34). Studies have shown that the Nrf2 level of RPE cells induced by H_2_O_2_ was significantly reduced, and the decreasing trend of Nrf2 was significantly reversed by phillyrin; the high expression of Keap1 was inhibited (35). Bovine mammary epithelial cell line MAC-T cells were induced by LPS, and the Nrf2 level significantly decreased. After treatment with *Dandelion* aqueous extract (DAE), the Nrf2 level significantly increased (36). Similarly, in this study, HS stimulation also reduced the level of Nrf2. After the ACP intervention, the low Nrf2 level caused by HS was reversed. HSP plays an important role in heat tolerance; its activation and the induced expression are positively correlated with heat tolerance. That is, exposure to extreme heat acts as a stimulus, triggering the activation and accumulation of HSP to protect functional proteins from degeneration (34). In the study, after 0,.5, and 2 h heat treatment, the level of HSP70 in the H9C2 cells was significantly higher than that in the normal temperature control group (30). This is consistent with our findings that HSP70 levels were significantly elevated after HS treatment and HSP70 levels were reversed to normal by ACP. These results suggest that the antioxidant effect of ACP may be related to the activation of Keap1-Nrf2.

The hypothalamus is vital to body temperature, feeding, drinking water, and energy regulation and is the center for regulating visceral and endocrine activities. The HPA axis plays an important role in stimulating and integrating various physiological and neural responses to adverse stimuli (14). The hypothalamus is an important component of the HPA axis and plays a key role in the balance of related hormone levels. The HPA axis is a major stress marker, and one of the most important results of its activation is the increase in the COR levels (37). When stimulated by high temperature, the hypothalamus integrates the body's physiological and neural responses to release CRH, which stimulates the pituitary gland to release ACTH and which acts on the adrenal gland and releases COR. COR can restrict part of the HPA axis through the negative feedback regulation mechanism to maintain the hormone level within the homeostasis range. A link is thought to exist between the brain and the gut, perhaps related to environmental factors or the gut microbiome, and growing number of evidence proves that the intestinal flora plays a key role (38–40). Changes in brain stress responses, sports, anxiety, and social behavior are thought to be linked to the gut microbiota (41–44). The vulnerability of the stress response network to maladaptive development was demonstrated by modeling animal models to explore the interaction between the activation of intestinal flora in the HPA axis of stress response and brain response (45). The HPA axis has long been thought to play a key role in regulating the balance of hormone secretion (14). Previous studies have shown that HS increases the level of COR in the serum tissue (23, 46). Our results are consistent with this. CRH and ACTH levels were also significantly increased in the HS group, suggesting that HS stimulates the regulation mechanism of the HPA axis. In addition, the levels of CRH, ACTH, and COR were significantly reduced to normal levels by ACP. It was found that the enrichment of intestinal flora in the HS environment was significantly different from that in the NHS group and the HS_ACP group, and some of the flora was closely related to the HPA axis related hormone levels. LEfSe and Spearman correlation analysis showed that g_UCG_005 significantly enriched in the HS group and promoted the secretion of ACTH and COR. *Barnesiella* was negatively correlated with ACTH and COR and had a higher LDA score in the HS_ACP group, suggesting that the alleviating effect of ACP on HS may be related to the activation of the HPA axis and intestinal flora. Wu et al. demonstrated that adrenal resection, glucocorticoid receptor antagonism, or COR synthesis inhibition can effectively correct social deficits after microbiome depletion (47).

The small intestine is the main site and the organ for digestion and absorption. The small intestinal villi are villous protrusions on the annular folds of the inner wall of the small intestine, which have the function of enlarging absorption surface area and filtering. The crypt is a depression on the intestinal wall; VH and CD are closely related to nutrient absorptive capacity, and the higher the ratio of VH and CD, the better the absorptive capacity. Instead, it shows possible pathological changes in the intestinal wall that can affect nutrient absorption (48). Extremely high ambient temperatures can have harmful effects on the intestines (49). Studies have shown that the ileum, as the end of the small intestine, is most vulnerable to HS (50). HS causes intestinal epithelial ischemia, impaired tight junction function, and changes in intestinal integrity (8); damage to the intestinal barrier increases the permeability of luminal antigens, leading to an inflammatory response, and stimulates the expression of pro-inflammatory cytokines (9, 10). In this study, we found that HS caused intestinal and systemic inflammation, and the level of pro-inflammatory cytokine IL-6 in the ileum and the serum significantly increased. Unfortunately, the secretion of anti-inflammatory cytokine IL-10 was inhibited. In addition, HS destroyed the integrity of the intestines and reduced the height of villi. SIgA plays a key role in intestinal mucosal immunity and is secreted in large quantities to balance mucosal immunity during inflammation. Intestinal mucosa is an important barrier to protect the internal environment of animals from microbiota and their secretions (51). Tight junctions are essential for intestinal barrier function and gastrointestinal permeability, with protein structures located in intercellular spaces between epithelial cells in the intestinal wall (52, 53). Several studies on poultry have shown that HS impairs intestinal integrity and increases pro-inflammatory cytokines (11, 54, 55). Our results are consistent with this. The levels of Occludin, Claudin-1, ZO-1, and IL-6 significantly reduced due to HS exposure. Previous studies have found that adding Chinese herbs to diets may reverse this adverse situation (18, 56). The adverse effects of HS on intestinal inflammatory factors and tight junction protein were repaired by ACP and tended to return to normal levels. Moreover, the VH and CD significantly increased, and the intestinal morphology was repaired. We found that ACP has a positive regulatory effect on intestinal immunity and the intestinal barrier.

In recent years, intestinal flora has become a research hotspot due to its ability to regulate digestion and absorption, neuroendocrinology, and so on, which is closely related to host growth performance, immunity, and stress response (57). HS can disrupt gut microbes (58, 59). In this study, at the phylum level, *Firmicutes* and *Bacteroidetes* dominated the cecal microflora of broilers. *Firmicutes* increased and *Bacteroidetes* decreased under HS (60). Consistent with our research results, *Firmicutes* and *Bacteroidetes* were the main microorganisms, and the number of *Firmicutes* increased significantly under HS, which was 25% higher than that of the control group. At the genus level, the level of *Alipipes* in the HS group was significantly higher than in the NHS group. Studies have shown that the adverse effects of cecal flora on host memory in chronic stress may be related to *Alipipes* (61). It can be seen from α diversity that the chao1 index of the HS group is higher than that of the NHS group. It is suspected that HS destroys the integrity of the intestines and facilitates the colonization of harmful bacteria in the intestines. Studies have shown that RES treatment reduces the negative impact of LPS on intestinal microbes by reducing the relative abundance of *Alipipes* and has a great potential in preventing inflammation (62). Our research results are consistent with this. The addition of ACP significantly reduces the relative abundance of *Alipipes* and improves the chao1 index. The results of LEfSe analysis showed that *Alipipes* was significantly negatively correlated with SOD and ZO-1 and was significantly positively correlated with IL-6 and MDA. This shows that *Alipipes* has a negative effect on the level of anti-oxidation, immunity, and tight junctions. Fortunately, the addition of ACP reversed the negative effects caused by HS. The Spearman correlation analysis showed that Occludin, claudin-1, and IL-10 were positively correlated with *Barnesiella* and were negatively correlated with *f_Rikenellaceae*, UCG_005*, f_Bacillaceae*. The correlation between the above flora and IL-6 is just the opposite. LEfSe analysis showed that the former was mainly enriched in the ACP supplement group, while the latter was mainly enriched in the HS group. This interesting finding suggests that the regulation of ACP on intestinal immunity and intestinal barrier may be related to intestinal flora.

## Conclusion

The aim of this study was to investigate the protective effects of ACP on oxidative stress, endocrine disorders, immune damage, and intestinal health damage in heat-stressed poultry. The results showed that ACP improved the antioxidant level, regulated endocrine disorder, and strengthened immunity. Enhancing intestinal health is a key pathway for ACP to regulate antioxidant and endocrine function in heat-stressed poultry. ACP effectively restored intestinal morphology and mucosal barrier integrity and improved intestinal microbial diversity and HS-induced intestinal flora disorder. In addition, it promoted the activation of Nrf2-related signaling pathway and the HPA axis. These results provide a theoretical basis for the development and application of ACP as a drug for alleviating HS in poultry and have potential value in anti-oxidation and regulating endocrine balance.

## Data Availability Statement

The datasets presented in this study can be found in online repositories. The names of the repository/repositories and accession number(s) can be found below: https://www.ncbi.nlm.nih.gov/, PRJNA790483.

## Ethics Statement

The animal study was reviewed and approved by Institutional Animal Care and Use Committee of Guangxi University (Approval No. 2021-165).

## Author Contributions

YiW, WS, and HS conceived the study and designed the project. YiW conducted the experiments, collected samples, analyzed data, and drafted the manuscript. YuW, FL, YC, and XP provided assistance in animal testing and sampling. KW, GZ, XC, and EW helped with the sampling and data analysis. WS and HS revised the manuscript and supervised the entire study. All authors contributed to this article and approved the submitted version.

## Funding

This study received funding from the Key Research and Development Plan of Guangxi, China (AB19245037), the Natural National Science Foundation of China (317607446), and the Major R&D Project of Wuming District Nanning China (20210111).

## Conflict of Interest

The authors declare that the research was conducted in the absence of any commercial or financial relationships that could be construed as a potential conflict of interest.

## Publisher's Note

All claims expressed in this article are solely those of the authors and do not necessarily represent those of their affiliated organizations, or those of the publisher, the editors and the reviewers. Any product that may be evaluated in this article, or claim that may be made by its manufacturer, is not guaranteed or endorsed by the publisher.

## Supplementary Material

Supplementary Figure 1 Experiment design.

Supplementary Figure 2 Rarefaction curve.

Supplementary Figure 3 Shannon-Wiener.

Supplementary Table 1 Ingredients of the experimental diets.

Supplementary Table 2 Abundances of dominant species at phyla, family, and genus levels.

## Abbreviations

HS: Heat stress
ACP: polysaccharide of *Abrus cantoniensis* Hance
VH: Villus height
CD: Crypt depth
IWT: Intestinal wall thickness
VH/CD: Villus height/Crypt depth
OTU: Operational taxonomic unit
SOD: Superoxide dismutase
MDA: Malonaldehyde
Nrf2: Nuclear factor erythroid-2 related factor 2
Keap1: Kelch-like ECH-associated protein 1
HSP70: Heat Shock Protein 70
CRH: Corticotropin releasing hormone
ACTH: Adrenocorticotropic hormone
COR: Corticosterone
IL-6: Interleukin 6
IL-10: Interleukin 10
ZO-1: Human tight junction protein 1
SIgA: Secretory IgA
VH: Villus height
CD: Crypt depth
IWT: Intestinal wall thickness.
