# Supplementary material for: Polysaccharides From Abrus cantoniensis Hance Modulate Intestinal Microflora and Improve Intestinal Mucosal Barrier and Liver Oxidative Damage Induced by Heat Stress
Source: Front Vet Sci. 2022 Apr 4;9:868433. doi: 10.3389/fvets.2022.868433 (PMC9013755; doi:10.3389/fvets.2022.868433)
Supplement: Supplementary Figure 1 — Experiment design. [file Data_Sheet_1.zip › Table S1. Ingredients of the experimental diets.docx]

**Table S1.** Ingredients of the experimental diets

Ingredients, %

CP 15.0-17.0

Crude fibre 25.0

Crude ash 30.0

Total P 0.3

Ca 0.6-1.5

Nacl 0.3-0.8

H2O 13

Methionine 0.2-0.7

Composition of main raw materials：Corn, wheat bran, Fishmeal, Soybean meal, Methionine, Vitamin A, Vitamin D3, Vitamin E, Vitamin B12, CaHPO4, CaCO3, Fe2SO4, CuSO4, ZnSO4, MnSO4, KI, Sodium selenite, NaCl, Soybean oil and so on.
