# Supplementary figures and images for: Polysaccharides From Abrus cantoniensis Hance Modulate Intestinal Microflora and Improve Intestinal Mucosal Barrier and Liver Oxidative Damage Induced by Heat Stress
Source: Front Vet Sci. 2022 Apr 4;9:868433. doi: 10.3389/fvets.2022.868433 (PMC9013755; doi:10.3389/fvets.2022.868433)

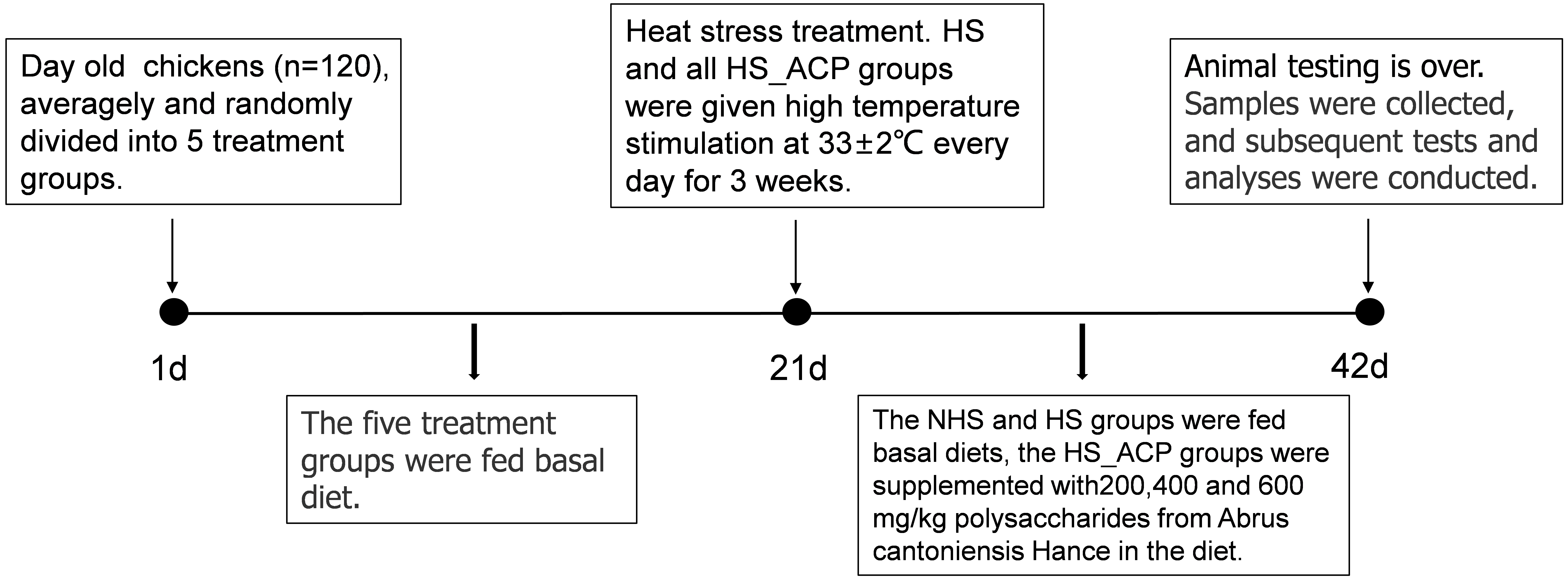

Supplement: Supplementary Figure 1 — Experiment design. [file Data_Sheet_1.zip › Supplementary Figure 1. Experiment design .tif]

# Multy samples Rarefaction Curves

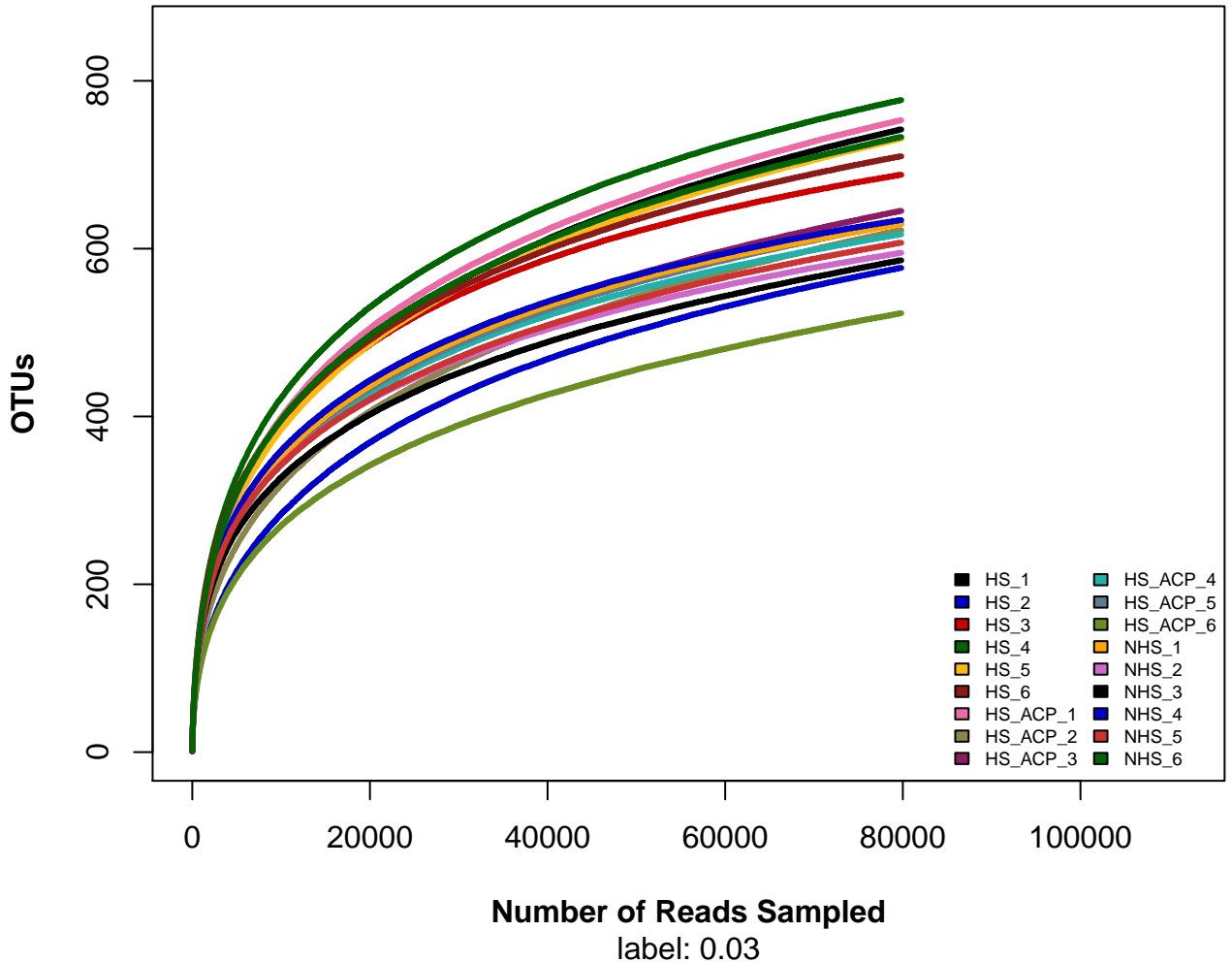

Supplement: Supplementary Figure 1 — Experiment design. [file Data_Sheet_1.zip › Supplementary Figure 2. Rarefaction curve.pdf]

# Multy samples Shannon–Wiener Curves

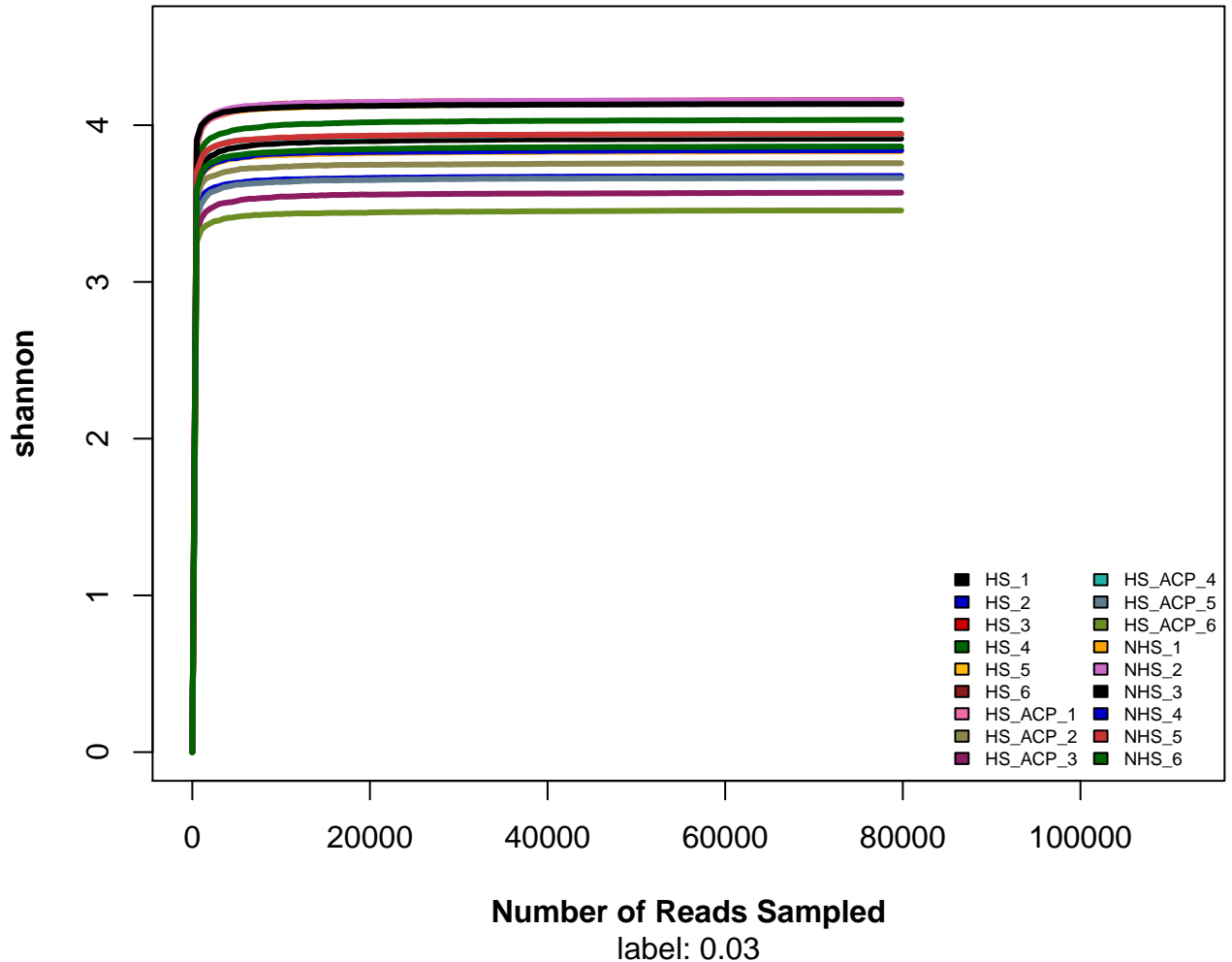

Supplement: Supplementary Figure 1 — Experiment design. [file Data_Sheet_1.zip › Supplementary Figure 3. Shannon-Wiener.pdf]
